# Methyl 5-hy­droxy-3-phenyl-1,2-oxazolidine-5-carboxyl­ate

**DOI:** 10.1107/S1600536812017576

**Published:** 2012-04-25

**Authors:** Jia Ye, Ya-Nan Liu, Yu Cheng

**Affiliations:** a College of Chemistry and Chemical Engineering, China West Normal University, Nanchong 637002, People’s Republic of China

## Abstract

In the title compound, C_11_H_13_NO_4_, the isoxazolidine ring has an envelope conformation with the O atom as the flap. In the crystal, mol­ecules are liked *via* N—H⋯O and bifurcated O—H⋯(O,N) hydrogen bonds forming chains propagating along [010]. There are also C—H⋯O inter­actions present.

## Related literature

For the use of isoxazolidine-containing compounds as building blocks in synthesis, see: Carrillo *et al.* (2006 ▶); Lv *et al.* (2010 ▶); Ibrahem *et al.* (2007 ▶); Sharma *et al.* (1999 ▶). For information on conjugation additions to α,β-unsaturated ketones, see: Wu *et al.* (2006 ▶). For standard bond-lengths see: Allen *et al.* (1987 ▶).
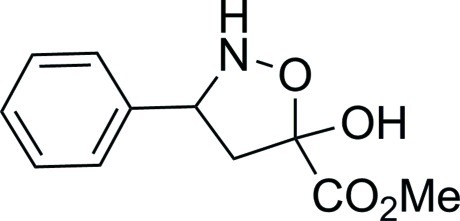


## Experimental

### 

#### Crystal data

C_11_H_13_NO_4_
*M* *_r_* = 223.22
Monoclinic, 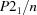

*a* = 11.8322 (3) Å
*b* = 6.0853 (1) Å
*c* = 15.8570 (3) Å
β = 101.864 (2)°
*V* = 1117.35 (4) Å^3^
*Z* = 4
Cu *K*α radiation
μ = 0.85 mm^−1^
*T* = 291 K
0.40 × 0.36 × 0.30 mm

#### Data collection

Oxford Gemini S Ultra diffractometer
Absorption correction: multi-scan (*CrysAlis PRO*; Oxford Diffraction, 2009 ▶) *T*_min_ = 0.726, *T*_max_ = 0.784
10901 measured reflections
2165 independent reflections
1921 reflections with *I* > 2σ(*I*)
*R*_int_ = 0.034

#### Refinement

*R*[*F* ^2^ > 2σ(*F* ^2^)] = 0.042
*wR*(*F* ^2^) = 0.097
*S* = 1.05
2165 reflections
151 parameters
H atoms treated by a mixture of independent and constrained refinement
Δρ_max_ = 0.20 e Å^−3^
Δρ_min_ = −0.12 e Å^−3^

### 

Data collection: *CrysAlis PRO* (Oxford Diffraction, 2009 ▶); cell refinement: *CrysAlis PRO*; data reduction: *CrysAlis PRO*; program(s) used to solve structure: *SHELXS97* (Sheldrick, 2008 ▶); program(s) used to refine structure: *SHELXL97* (Sheldrick, 2008 ▶); molecular graphics: *ORTEP-3* (Farrugia, 1997 ▶); software used to prepare material for publication: *SHELXL97*.

## Supplementary Material

Crystal structure: contains datablock(s) global, I. DOI: 10.1107/S1600536812017576/su2406sup1.cif

Structure factors: contains datablock(s) I. DOI: 10.1107/S1600536812017576/su2406Isup2.hkl

Supplementary material file. DOI: 10.1107/S1600536812017576/su2406Isup3.cml

Additional supplementary materials:  crystallographic information; 3D view; checkCIF report

## Figures and Tables

**Table 1:** Hydrogen-bond geometry (Å, °)

*D*—H⋯*A*	*D*—H	H⋯*A*	*D*⋯*A*	*D*—H⋯*A*
N1—H4⋯O1^i^	0.907 (18)	2.315 (18)	3.1158 (15)	147.0 (13)
O2—H9⋯O1^i^	0.82	2.51	3.0673 (13)	127
O2—H9⋯N1^i^	0.82	1.99	2.7826 (16)	162
C11—H11*A*⋯O3^ii^	0.96	2.54	3.480 (2)	166
C11—H11*C*⋯O2^iii^	0.96	2.53	3.419 (2)	154

Symmetry codes: (i) 
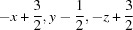
; (ii) 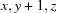; (iii) 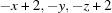.

**Fig. 1.**
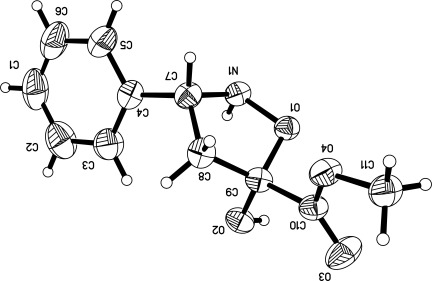
The molecular structure of the title molecule, with the atom numbering. Displacement ellipsoids are drawn at the 30% probability level.

## supplementary crystallographic information

### Comment

Isoxazolidines are interesting heterocyclic compounds that may be regarded as unusual constrained β-amino acids or as furanose mimetics, and have been exploited as analogues of natural products (Lv et al., 2010). Isoxazolidines are also applied in the synthesis of oligopeptides in the absence of coupling reagents (Carrillo et al., 2006) and used as building blocks in organic synthesis (Ibrahem et al., 2007; Sharma et al., 1999).

Nitrogen containing nucleophiles such as hydroxylamines and hydrazoic acid are widely employed in conjugation additions to α,β-unsaturated ketones (Wu et al., 2006). The title compound is a Michael addition product from the transformation of hydroxylamine to an α,β-unsaturated ketone ester. We report herein on the crystal structure of the title compound.

The molecular structure of the title molecule is shown in Fig. 1. The bond lengths (Allen et al., 1987) and angles are normal. The isoxazolidine ring possesses an envelope conformation with atom O1 as the flap.

In the crystal, molecules are linked via N—H···O and bifurcated O—H···O,N hydrogen bonds to form chains along the b axis (Table 1). These chains are linked via C-H···O interactions (Table 1).

### Experimental

To the solution of (*E*)-methyl 2-oxo-4-phenylbut-3-enoate (0.019 g, 0.1 mmol) and hydroxylamine hydrochloride (0.07 g, 0.1 mmol) in dichloromethane (1 mL) was added triethylamine (0.012 g, 0.12mmol) at room temperature. The reaction mixture was stirred for 24 h at 273 K. The solvent was then removed under reduced pressure, and the residue was purified through column chromatography (petroleum ether: ethyl acetate = 3:1(V/V)). Single crystals, suitable for X-ray diffraction, were obtained by slow evaporation of an ethyl acetate solution at room temperature for 2 d.

### Refinement

The NH H atom was located in a difference Fourier map and freely refined. The OH and C-bound H-atoms were included in calculated positions and treated as riding atoms: O-H = 0.82 Å, C-H = 0.93, 0.96, 0.97 and 0.98 Å for CH(aromatic), CH_3_, CH_2_ and CH(methine) H-atoms, respectively, with U_iso_(H) = k × U_eq_(O,C), where k = 1.5 for OH and CH_3_ H-atoms and = 1.2 for other H-atoms.

### Crystal data

**Table:**

C_11_H_13_NO_4_	*F*(000) = 472
*M**_r_* = 223.22	*D*_x_ = 1.327 Mg m^−^^3^
Monoclinic, *P*2_1_/*n*	Cu *K*α radiation, λ = 1.54184 Å
Hall symbol: -P 2yn	Cell parameters from 7888 reflections
*a* = 11.8322 (3) Å	θ = 3.8–71.9°
*b* = 6.0853 (1) Å	µ = 0.85 mm^−^^1^
*c* = 15.8570 (3) Å	*T* = 291 K
β = 101.864 (2)°	Block, colourless
*V* = 1117.35 (4) Å^3^	0.40 × 0.36 × 0.30 mm
*Z* = 4	

### Data collection

**Table:**

Oxford Gemini S Ultra diffractometer	2165 independent reflections
Radiation source: Enhance Ultra (Cu) X-ray Source	1921 reflections with *I* > 2σ(*I*)
Mirror monochromator	*R*_int_ = 0.034
Detector resolution: 15.9149 pixels mm^-1^	θ_max_ = 72.1°, θ_min_ = 5.7°
ω scans	*h* = −14→13
Absorption correction: multi-scan (*CrysAlis PRO*; Oxford Diffraction, 2009)	*k* = −6→7
*T*_min_ = 0.726, *T*_max_ = 0.784	*l* = −19→18
10901 measured reflections	

### Refinement

**Table:**

Refinement on *F*^2^	Primary atom site location: structure-invariant direct methods
Least-squares matrix: full	Secondary atom site location: difference Fourier map
*R*[*F*^2^ > 2σ(*F*^2^)] = 0.042	Hydrogen site location: inferred from neighbouring sites
*wR*(*F*^2^) = 0.097	H atoms treated by a mixture of independent and constrained refinement
*S* = 1.05	*w* = 1/[σ^2^(*F*_o_^2^) + (0.0323*P*)^2^ + 0.2933*P*] where *P* = (*F*_o_^2^ + 2*F*_c_^2^)/3
2165 reflections	(Δ/σ)_max_ < 0.001
151 parameters	Δρ_max_ = 0.20 e Å^−^^3^
0 restraints	Δρ_min_ = −0.12 e Å^−^^3^

### Special details

**Table:**

Experimental. Absorption correction: (CrysAlisPro; Oxford Diffraction, 2009) Empirical absorption correction using spherical harmonics, implemented in SCALE3 ABSPACK scaling algorithm.
Geometry. Bond distances, angles etc. have been calculated using the rounded fractional coordinates. All su's are estimated from the variances of the (full) variance-covariance matrix. The cell esds are taken into account in the estimation of distances, angles and torsion angles
Refinement. Refinement of *F*^2^ against ALL reflections. The weighted *R*-factor *wR* and goodness of fit *S* are based on *F*^2^, conventional *R*-factors *R* are based on *F*, with *F* set to zero for negative *F*^2^. The threshold expression of *F*^2^ > σ(*F*^2^) is used only for calculating *R*-factors(gt) *etc*. and is not relevant to the choice of reflections for refinement. *R*-factors based on *F*^2^ are statistically about twice as large as those based on *F*, and *R*- factors based on ALL data will be even larger.

### Fractional atomic coordinates and isotropic or equivalent isotropic displacement parameters (Å^2^)

**Table:**

	*x*	*y*	*z*	*U*_iso_*/*U*_eq_	
O1	0.79693 (8)	0.06822 (15)	0.78232 (6)	0.0518 (3)	
O2	0.82481 (9)	−0.20986 (16)	0.88556 (6)	0.0604 (4)	
O3	1.04065 (10)	−0.0305 (2)	0.89858 (10)	0.0901 (5)	
O4	0.96722 (8)	0.30583 (16)	0.89779 (7)	0.0641 (4)	
N1	0.67179 (10)	0.0582 (2)	0.76513 (8)	0.0535 (4)	
C1	0.3265 (2)	−0.1664 (6)	0.87113 (15)	0.1076 (12)	
C2	0.4303 (2)	−0.2651 (4)	0.90278 (16)	0.1061 (10)	
C3	0.53265 (17)	−0.1617 (3)	0.89530 (14)	0.0857 (7)	
C4	0.53083 (13)	0.0405 (3)	0.85666 (10)	0.0629 (5)	
C5	0.42474 (15)	0.1361 (4)	0.82446 (13)	0.0843 (7)	
C6	0.32309 (17)	0.0312 (5)	0.83205 (16)	0.1066 (10)	
C7	0.63963 (12)	0.1531 (2)	0.84333 (10)	0.0577 (5)	
C8	0.74876 (13)	0.1335 (3)	0.91548 (10)	0.0613 (5)	
C9	0.83371 (12)	0.0138 (2)	0.87205 (9)	0.0522 (4)	
C10	0.96003 (12)	0.0898 (2)	0.89183 (9)	0.0553 (5)	
C11	1.08082 (14)	0.4029 (3)	0.90958 (12)	0.0719 (6)	
H1	0.25820	−0.23540	0.87650	0.1290*	
H2	0.43250	−0.40170	0.92940	0.1270*	
H3	0.60300	−0.23020	0.91670	0.1030*	
H4	0.6564 (13)	−0.088 (3)	0.7630 (10)	0.062 (4)*	
H5	0.42140	0.27220	0.79740	0.1010*	
H6	0.25230	0.09750	0.81010	0.1280*	
H7	0.62260	0.30950	0.83270	0.0690*	
H8A	0.77780	0.27740	0.93560	0.0740*	
H8B	0.73310	0.04990	0.96390	0.0740*	
H9	0.84150	−0.27740	0.84510	0.0900*	
H11A	1.07520	0.55870	0.91730	0.1080*	
H11B	1.11280	0.37440	0.85970	0.1080*	
H11C	1.12990	0.34020	0.95950	0.1080*	

### Atomic displacement parameters (Å^2^)

**Table:**

	*U*^11^	*U*^22^	*U*^33^	*U*^12^	*U*^13^	*U*^23^
O1	0.0502 (5)	0.0495 (6)	0.0538 (5)	−0.0012 (4)	0.0064 (4)	0.0012 (4)
O2	0.0731 (7)	0.0478 (6)	0.0577 (6)	−0.0060 (5)	0.0076 (5)	0.0027 (4)
O3	0.0607 (7)	0.0555 (7)	0.1493 (12)	0.0081 (6)	0.0105 (7)	−0.0051 (7)
O4	0.0539 (6)	0.0465 (6)	0.0867 (8)	−0.0031 (4)	0.0023 (5)	0.0018 (5)
N1	0.0496 (6)	0.0472 (7)	0.0608 (7)	−0.0018 (5)	0.0049 (5)	−0.0032 (5)
C1	0.0765 (14)	0.163 (3)	0.0888 (15)	−0.0423 (16)	0.0295 (11)	−0.0271 (16)
C2	0.1073 (18)	0.1077 (18)	0.1067 (16)	−0.0414 (14)	0.0303 (13)	0.0013 (13)
C3	0.0734 (11)	0.0809 (13)	0.1028 (14)	−0.0142 (10)	0.0179 (10)	0.0070 (11)
C4	0.0565 (8)	0.0657 (10)	0.0669 (9)	−0.0030 (7)	0.0136 (7)	−0.0112 (8)
C5	0.0627 (10)	0.0975 (14)	0.0922 (13)	0.0080 (10)	0.0149 (9)	−0.0081 (11)
C6	0.0572 (11)	0.153 (2)	0.1111 (18)	−0.0031 (13)	0.0211 (11)	−0.0198 (17)
C7	0.0593 (8)	0.0481 (8)	0.0653 (9)	0.0006 (6)	0.0120 (7)	−0.0059 (6)
C8	0.0593 (8)	0.0635 (9)	0.0605 (8)	−0.0063 (7)	0.0110 (7)	−0.0115 (7)
C9	0.0564 (8)	0.0463 (7)	0.0511 (7)	−0.0023 (6)	0.0046 (6)	−0.0007 (6)
C10	0.0566 (8)	0.0474 (8)	0.0588 (8)	0.0015 (6)	0.0044 (6)	0.0008 (6)
C11	0.0593 (9)	0.0615 (10)	0.0893 (12)	−0.0118 (8)	0.0026 (8)	0.0030 (8)

### Geometric parameters (Å, º)

**Table:**

O1—N1	1.4507 (16)	C5—C6	1.389 (3)
O1—C9	1.4384 (17)	C7—C8	1.544 (2)
O2—C9	1.3852 (16)	C8—C9	1.517 (2)
O3—C10	1.1897 (18)	C9—C10	1.534 (2)
O4—C10	1.3195 (16)	C1—H1	0.9300
O4—C11	1.445 (2)	C2—H2	0.9300
O2—H9	0.8200	C3—H3	0.9300
N1—C7	1.4866 (19)	C5—H5	0.9300
N1—H4	0.907 (18)	C6—H6	0.9300
C1—C2	1.367 (4)	C7—H7	0.9800
C1—C6	1.350 (5)	C8—H8A	0.9700
C2—C3	1.391 (3)	C8—H8B	0.9700
C3—C4	1.373 (3)	C11—H11A	0.9600
C4—C7	1.511 (2)	C11—H11B	0.9600
C4—C5	1.383 (3)	C11—H11C	0.9600
			
N1—O1—C9	105.41 (10)	O4—C10—C9	111.15 (11)
C10—O4—C11	117.46 (12)	C2—C1—H1	120.00
C9—O2—H9	109.00	C6—C1—H1	120.00
O1—N1—C7	104.68 (10)	C1—C2—H2	120.00
C7—N1—H4	109.2 (10)	C3—C2—H2	120.00
O1—N1—H4	103.7 (10)	C2—C3—H3	120.00
C2—C1—C6	120.0 (2)	C4—C3—H3	120.00
C1—C2—C3	120.2 (2)	C4—C5—H5	120.00
C2—C3—C4	120.62 (19)	C6—C5—H5	120.00
C3—C4—C5	118.15 (17)	C1—C6—H6	120.00
C3—C4—C7	122.23 (15)	C5—C6—H6	120.00
C5—C4—C7	119.50 (16)	N1—C7—H7	108.00
C4—C5—C6	120.8 (2)	C4—C7—H7	108.00
C1—C6—C5	120.3 (2)	C8—C7—H7	108.00
C4—C7—C8	117.92 (13)	C7—C8—H8A	111.00
N1—C7—C8	105.64 (11)	C7—C8—H8B	111.00
N1—C7—C4	108.18 (12)	C9—C8—H8A	111.00
C7—C8—C9	103.42 (12)	C9—C8—H8B	111.00
O1—C9—O2	111.23 (10)	H8A—C8—H8B	109.00
O1—C9—C8	104.16 (11)	O4—C11—H11A	109.00
C8—C9—C10	118.11 (12)	O4—C11—H11B	109.00
O1—C9—C10	102.52 (11)	O4—C11—H11C	109.00
O2—C9—C8	108.85 (12)	H11A—C11—H11B	109.00
O2—C9—C10	111.50 (11)	H11A—C11—H11C	109.00
O3—C10—O4	124.61 (14)	H11B—C11—H11C	109.00
O3—C10—C9	124.22 (12)		
			
C9—O1—N1—C7	38.90 (12)	C3—C4—C7—C8	−38.7 (2)
N1—O1—C9—O2	76.12 (13)	C5—C4—C7—N1	−94.88 (18)
N1—O1—C9—C8	−40.98 (12)	C5—C4—C7—C8	145.43 (16)
N1—O1—C9—C10	−164.61 (9)	C4—C5—C6—C1	0.0 (4)
C11—O4—C10—O3	2.8 (2)	N1—C7—C8—C9	−3.24 (15)
C11—O4—C10—C9	−175.29 (12)	C4—C7—C8—C9	117.76 (14)
O1—N1—C7—C4	−148.04 (11)	C7—C8—C9—O1	26.43 (14)
O1—N1—C7—C8	−20.90 (13)	C7—C8—C9—O2	−92.30 (13)
C6—C1—C2—C3	0.4 (4)	C7—C8—C9—C10	139.28 (12)
C2—C1—C6—C5	−0.6 (4)	O1—C9—C10—O3	−103.08 (16)
C1—C2—C3—C4	0.4 (3)	O1—C9—C10—O4	75.06 (13)
C2—C3—C4—C5	−1.0 (3)	O2—C9—C10—O3	16.0 (2)
C2—C3—C4—C7	−176.90 (18)	O2—C9—C10—O4	−165.86 (11)
C3—C4—C5—C6	0.8 (3)	C8—C9—C10—O3	143.17 (16)
C7—C4—C5—C6	176.83 (19)	C8—C9—C10—O4	−38.69 (17)
C3—C4—C7—N1	81.02 (19)		

### Hydrogen-bond geometry (Å, º)

**Table:**

*D*—H···*A*	*D*—H	H···*A*	*D*···*A*	*D*—H···*A*
N1—H4···O1^i^	0.907 (18)	2.315 (18)	3.1158 (15)	147.0 (13)
O2—H9···O1^i^	0.82	2.51	3.0673 (13)	127
O2—H9···N1^i^	0.82	1.99	2.7826 (16)	162
C11—H11*A*···O3^ii^	0.96	2.54	3.480 (2)	166
C11—H11*C*···O2^iii^	0.96	2.53	3.419 (2)	154

Symmetry codes: (i) −*x*+3/2, *y*−1/2, −*z*+3/2; (ii) *x*, *y*+1, *z*; (iii) −*x*+2, −*y*, −*z*+2.
